# Edge chlorination of hexa-*peri*-hexabenzocoronene investigated by density functional theory and vibrational spectroscopy†
†Electronic supplementary information (ESI) available: Description and animations of the vibrational normal modes of HBC and HBC-Cl discussed in the text. See DOI: 10.1039/c5cp07755a



**DOI:** 10.1039/c5cp07755a

**Published:** 2016-02-25

**Authors:** Ali Maghsoumi, Akimitsu Narita, Renhao Dong, Xinliang Feng, Chiara Castiglioni, Klaus Müllen, Matteo Tommasini

**Affiliations:** a Dipartimento di Chimica , Materiali e Ingegneria Chimica – Politecnico di Milano , Piazza Leonardo da Vinci , 32-20133 Milano , Italy . Email: matteo.tommasini@polimi.it; b Max Planck Institute for Polymer Research , Ackermannweg 10 , D-55128 , Mainz , Germany . Email: muellen@mpip-mainz.mpg.de; c Center for Advancing Electronics Dresden (CFAED) , Department of Chemistry and Food Chemistry , Dresden University of Technology , Walther-Hempel-Bau Mommsenstrasse 4 , 01062 Dresden , Germany

## Abstract

We investigate the molecular structure and vibrational properties of perchlorinated hexa-*peri*-hexabenzocoronene (HBC-Cl) by density functional theory (DFT) calculations and IR and Raman spectroscopy, in comparison to the parent HBC. The theoretical and experimental IR and Raman spectra demonstrated very good agreement, elucidating a number of vibrational modes corresponding to the observed peaks. Compared with the parent HBC, the edge chlorination significantly alters the planarity of the molecule. Nevertheless, the results indicated that such structural distortion does not significantly impair the π-conjugation of such polycyclic aromatic hydrocarbons.

## Abstract 2

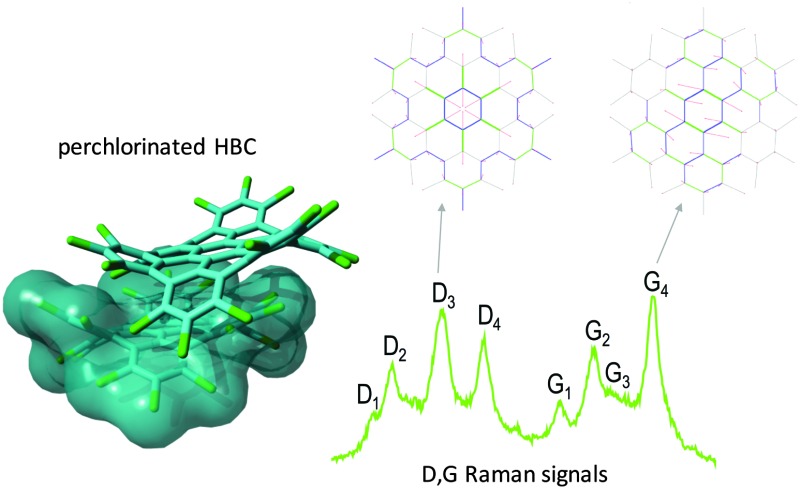
The molecular structure and vibrational properties of perchlorinated HBC and the parent HBC have been investigated by density functional theory calculations and vibrational spectroscopy.

## 1 Introduction

**Fig. 1:**
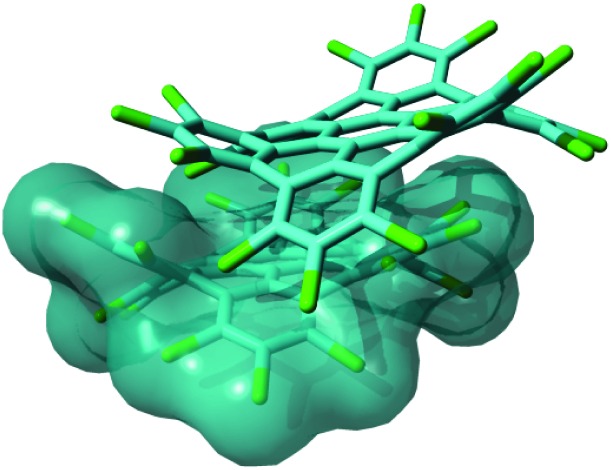
Representation of a π stacked dimer of HBC-Cl obtained from geometry optimization using DFT methods including Grimme's dispersion (B3LYP/6-31G(d,p) GD3BJ).^10^ This structure is very similar to the one determined by X-ray diffraction.^3^

Perchlorination of extended polycyclic aromatic hydrocarbons (PAHs) has recently been developed as a convenient way not only to modulate the optical properties,^1^ or the formation of self-assembled monolayers,^2^ but also to improve the solubility of such disk-shaped molecules,^3^ which can also be considered as molecularly defined model systems of graphene, *i.e.*, graphene molecules.^4,5^ Solubility is a technology-enabling key concept in view of the use of such π-conjugated molecules in real devices. The mechanism responsible for the enhancement of the solubility in perchlorinated graphene molecules was identified as an evident structural distortion of the molecules driven by steric hindrance of the chloro substituents at the molecular edges.^3^ This structural effect was shown to interfere with the well-known π-stacking propensity of such large PAHs, hence boosting their solubility.^3^ In this work we specifically address the case of perchlorinated hexa-*peri*-hexabenzocoronene (HBC-Cl). HBC is one of the prototypical PAHs that are able to form columnar arrays in discotic liquid crystals,^6^ which constitute relevant supramolecular architectures appealing for applications in molecular electronics.^7,8^ The packing of HBC-Cl in its crystalline form clearly reveals the presence of cofacial dimers,^3^ with properly interlocked orientation due to the accommodation of the out-of-plane deviations of the aromatic core of the molecule. This kind of interaction geometry is not merely the result of packing effects in the crystalline phase. DFT calculations on the isolated dimer of HBC-Cl straightforwardly account for this kind of molecular arrangement as shown in Fig. 1. Hence the cofacial self-assembly of HBC-Cl is essentially driven by interactions at the intermolecular level. The dimer shown in Fig. 1 can be also used as a model to evaluate the transfer integral (electronic coupling *t*)^9^ which is one of the relevant physical parameters on the basis of the appealing charge transport properties of graphene molecules, and determines their suitability for molecular electronics.^8^ An approximate *t* value can be easily obtained in the framework of the so-called Energy-Splitting-in Dimer Method.^9^ It considers how the doubly degenerate HOMO and LUMO levels of isolated HBC-Cl split and spread into the four occupied and four unoccupied frontier orbitals of the dimer. By evaluating half the energy bandwidth over which the four occupied (unoccupied) levels of the dimer spread out one gets *t* = 0.04 eV for holes (*t* = 0.03 eV for electrons). Interestingly, these values are located at the lower limit of the expected range found in other graphenic molecules with planar molecular shapes.^8^ This shows that perchlorination and the non-planarity of the molecule are not suppressing the charge transport properties: by suitable tuning of the relative molecular disposition and design of the chlorination pattern one could possibly enhance the value of *t*, which is known to dramatically depend on even small changes of the relative orientation angle and relative sliding of the aromatic cores.^8,9^

Intrigued by the appealing properties of HBC-Cl and its non-planar molecular structure we have carried out a joint experimental and theoretical study of the vibrational properties and the structure of HBC-Cl, adopting IR, Raman and DFT methods.

## 2 Results and discussion

### 2.1 Molecular structure of perchlorinated HBC

**Fig. 2:**
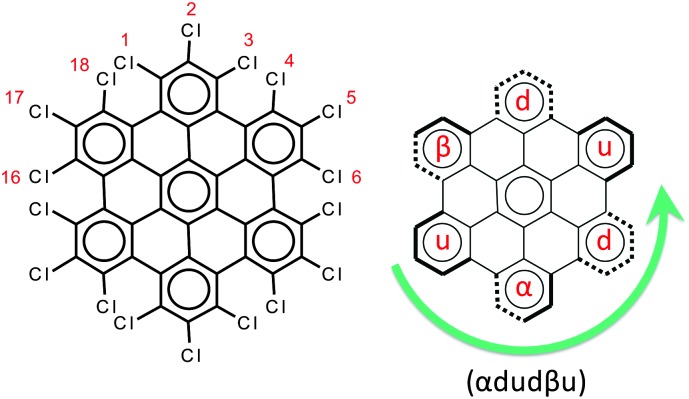
Left panel: The molecular structure of HBC-Cl. Right panel: The notation scheme for the possible out-of-plane conformations at the chlorinated edge (see the text for details).

The chemical structure of perchlorinated HBC (HBC-Cl) is shown in Fig. 2. While the parent HBC is a planar π-conjugated system,^11^ substitution with chlorine atoms at the edge introduces significant steric hindrance at the bay positions such as 1–18 (see Fig. 2). For this reason the equilibrium structure of HBC-Cl significantly deviates from planarity in order to increase the Cl–Cl distances at 1–18 and other bay positions (*e.g.*, 3–4). On the other hand, because of the larger Cl–Cl distances involving the substitutions at positions 1, 2 and 3, we may consider the chlorinated aryl moieties at the edge carbons as locally planar. This is a first approximation useful to simplify the notation for describing the possible out-of-plane conformations. Depending on the position of a given edge aryl moiety with respect to the average molecular plane, we may have four possible conditions, which are exemplified in Fig. 2:

• up (or down) if the aryl moiety lies above (or below) the plane;

• α (or β) if the aryl moiety lies in a propeller blade fashion, with α being related to *P*-helicity.

Because of steric hindrance (see Fig. 2) the conformation sequence cannot contain pairs such uu, dd, αβ, βα, uβ, dα, αu, and βd. The exhaustive enumeration of all possible combinations satisfying this prescription, considering the equivalence between enantiomeric pairs, leads to the eight conformers listed in Table 1. These conformers have been considered for geometry optimization using the DFT method in order to obtain information on their relative energies, which are also reported in Table 1. When α or β symbols are present in the structure, it is possible to have enantiomeric pairs. For the sake of compactness, for each enantiomeric pair in Table 1 we report the representative with a number of α symbols greater than the number of β symbols (this does not apply to conformers #3 and #7 which have the same number of α and β symbols). Obviously the conformation symbols can be cyclically permuted without changing the nature of the conformation. They can also be subjected to mirror symmetry with respect to the average molecular plane, *i.e.* α → β, u → d, and so on. This operation exchanges with one another the members of one enantiomeric pair, which of course possesses the same relative energy. Hence the conformation shown in Fig. 2, namely (αdudβu), by cyclic permutation can be transformed to (dudβuα) and by mirror symmetry is transformed to (uduαdβ), which corresponds to conformer #3 in Table 1.

**Table 1:** The description of the eight conformers of HBC-Cl and their relative energies (kcal mol^–1^) as determined by DFT calculations

#	Description	Short form	*S*	Energy	Group
1	(ududud)	(ud)_3_	12	0.0	*D*_3d_

2	(uαduαd)	(uαd)_2_	8	4.6	*D*_2_
3	(uduαdβ)	—	8	6.5	*C*_s_
4	(αduduα)	*α*_2_(du)_2_	8	8.9	*C*_2_

5	(ααααdu)	(α4du)	4	17.3	*C*_2_
6	(αααdβu)	(α3dβu)	4	19.1	*C*_2_
7	(ααdββu)	(α2dβ2u)	4	22.8	*C*_2h_

8	(αααααα)	(α6)	0	28.2	*D*_6_

**Fig. 3:**
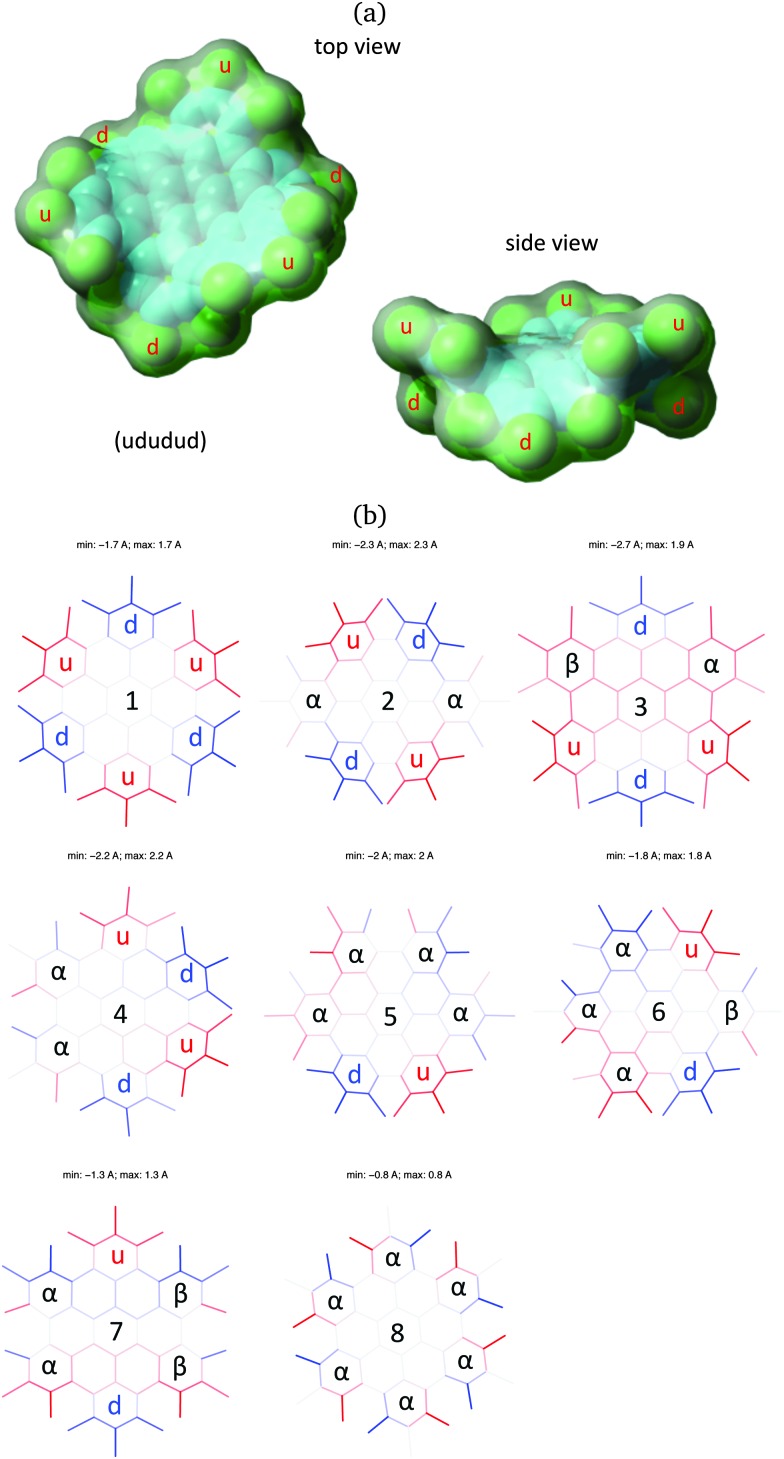
(a) Three-dimensional representation of (ud)_3_, the most stable conformer of HBC-Cl. The model represents the equilibrium structure obtained from DFT calculations. (b) Orthogonal projection of the eight conformations of HBC-Cl where the out-of-plane *z*-coordinate of the middle point of each bond is coded in shades of red (*z* > 0) and blue (*z* < 0). In-plane bonds are coded with light gray shade.

Interestingly, the most stable conformation of HBC-Cl corresponds to the only one non-chiral conformation. This is (ududud) ≡ (ud)_3_, which belongs to point group symmetry *D*_3d_ (see Fig. 3). In contrast (α_6_), the conformation with more extensive chirality (point group symmetry *D*_6_) is predicted to be the one with highest relative energy. Inspection of the relative energies of HBC-Cl conformations reported in Table 1 reveals an approximate correlation of their stability with the molecular structure at the edge, as described by the conformation symbol. We can introduce a score number *S* defined as follows:

1 *S* = *s*_1_*N*_1_ + *s*_2_*N*_2_ + *s*_3_*N*_3_

where *N*_1_ represents the number of ud (or du) sequences along the conformation string, considered in a cyclic manner, so that *N*_1_ = 6 for (ud)_3_. Similarly, *N*_2_ represents the total number of uα, αd, dβ, βu sequences along the string and *N*_3_ is the total number of αα, ββ sequences along the string. *s*_1_, *s*_2_, and *s*_3_ are suitable numerical coefficients which weight the different stability of the possible local edge conformation. The choice made in Table 1 simply assumes *s*_1_ = 2, *s*_2_ = 1 and *s*_3_ = 0, so that higher scores are associated with higher molecular stabilities. This effectively allows highlighting in Table 1 clusters of conformations characterized by similar energies and the same score number *S*.

After the evaluation of the stable conformations of HBC-Cl by means of the analysis of the DFT results, we can conclude that the most stable structure is fully consistent with experimental observation by X-ray diffraction, which clearly reveals (ud)_3_ as the unique structure of HBC-Cl in the crystal phase.^3^ This is expected based on the relatively large energy separation (4.6 kcal mol^–1^) of (ud)_3_ with the second most stable conformer, (uαd)_2_. We can conclude on this basis that the packing motif observed in the crystal simoultaneusly minimizes the intramolecular (conformational) energy and allows an effective intermolecular packing in the dimer.

Functionalisation of HBC with Cl at the molecular edge drives the molecule out of planarity, with a rich possibility of conformations, even though (ud)_3_ is markedly more stable than all the others (see Table 1). In principle out-of-plane distortions could negatively affect π-conjugation. Notably this is observed in π-conjugated polymers possessing torsional degrees of freedom able to affect the nearest neighbor π-interactions.^12^ Hence, to dwell more on the effects of distortion from planarity, we have considered a molecular model with the same conformation of the aromatic core as in (ud)_3_, but with a hydrogen-terminated molecular edge (the hydrogen positions have been fully optimized while keeping the position of the carbon atoms frozen at the positions they have in perchlorinated (ud)_3_). For simplicity we name this model HBC*. In addition to this model, we consider also HBC^†^, which is obtained starting from the structure of HBC* and fully optimizing all internal coordinates except dihedral angles. This effectively allows us to maintain the characteristic curved shape found in (ud)_3_, while fully relaxing the bond lengths and valence angles. The energy difference Δ*E* between the total energy of HBC*, HBC^†^ and HBC (see Table 2) is a measure of the energy cost associated with the distortion of the aromatic core from planarity. By considering the number of π-conjugated carbon atoms in the HBC analogues (42) this results in about 1.3 kcal mol^–1^ per carbon, a rather low value which explains the good stability of the compound despite its seeming dramatic distortion from planarity. Turning now to the electronic properties, we observe in Table 2 that along the sequence HBC, HBC*, HBC^†^ the position of the frontier orbitals does not change dramatically, which enforces the idea that π-conjugation is not seriously affected by the deviation of HBC from planarity. On the other hand perchlorination causes the decrease of the position of the frontier orbitals, as expected from an electron-withdrawing substitution. We notice that, compared to HBC*, in HBC-Cl the position of the HOMO (decrease by 0.04 ha) is relatively less affected than the position of the LUMO (decrease by 0.05 ha), which explains the slight decrease of the HOMO–LUMO gap in HBC-Cl. Following the trend of the position and spacing between the frontier orbitals, according to TDDFT calculations the low-lying doubly degenerate bright state red shifts from 3.47 eV (357 nm) in HBC to 2.98 eV (415 nm) in HBC-Cl, while the total oscillator strength of the doublet slightly increases from *f* = 1.44 to *f* = 1.54, respectively.

**Table 2:** Comparison of the relative positions of frontier orbitals (HOMO and LUMO) and of the lowest lying bright excited state (absorption maximum *λ*_max_) in planar HBC, distorted HBC*, HBC^†^ and HBC-Cl in its more stable conformation. Δ*E* is the energy difference between the non-planar models HBC* and HBC^†^ and planar HBC

	HBC	HBC^†^	HBC*	HBC-Cl (ud)_3_
Δ*E* (kcal mol^–1^)	0	53	54	—
HOMO (hartree)	–0.1929	–0.1898	–0.1897	–0.2316
LUMO (hartree)	–0.061	–0.0618	–0.0626	–0.115
*Δ*_HL_ (hartree)	0.132	0.128	0.127	0.117
*λ*_max_ (nm)	357	372	375	415
Δ*λ* (nm)	0	15	18	58

**Fig. 4:**
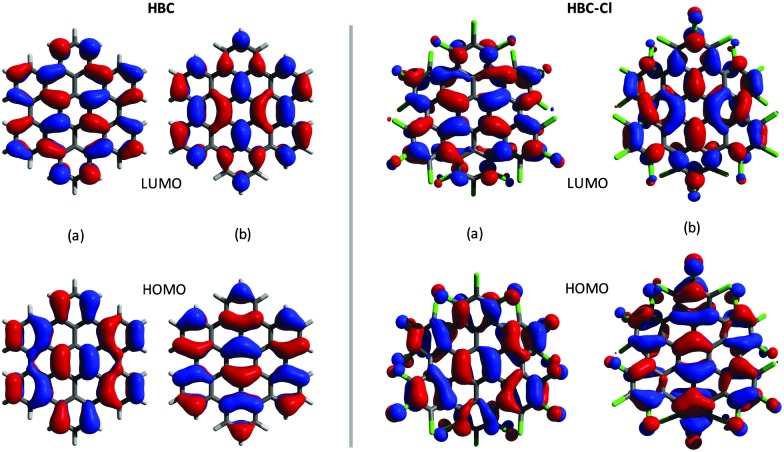
Representation of the HOMO and the LUMO of HBC (left (a and b) panels) and HBC-Cl (right (a and b) panels).

The representation of the doubly degenerate HOMO and LUMO of HBC-Cl and HBC is reported in Fig. 4. For both degenerate HOMO and LUMO pairs (a and b) represented in Fig. 4 it is possible to observe the close similarity existing between HBC-Cl and HBC. This further supports the conclusion that, despite the marked out-of-plane distortion, π-conjugation in HBC-Cl extends over the whole aromatic core of the molecule. This is also consistent with a recent report which highlights the remarkable non-linear optical properties of several non-planar chlorinated graphene molecules, including HBC-Cl.^1^

**Fig. 5:**
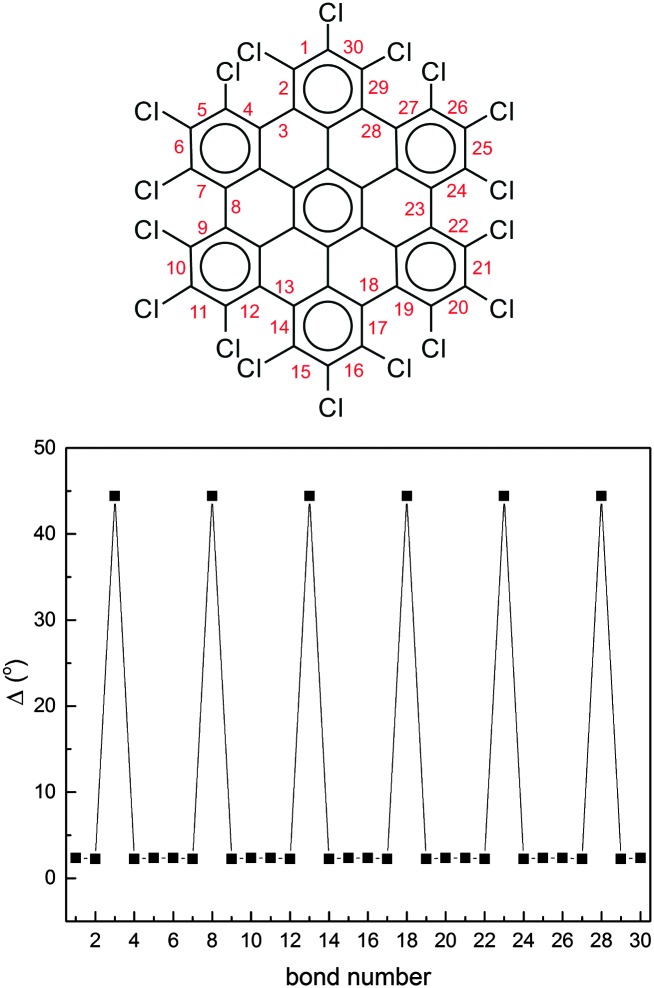
The deviation from planarity *Δ* of the dihedral angles at the molecular edge of HBC-Cl in the (ud)_3_ conformation obtained from DFT calculations. The definition of *Δ* is given in the text.

To further characterize the deviation from planarity in HBC-Cl it is useful to introduce the parameter *Δ*, given by the absolute value of the difference of a CCCC dihedral angle *τ* along the edge of the molecule from the *trans* or *cis* conformation expected in the planar case, *i.e.*:

2 *Δ* = |*τ* – 180°| (*trans*);

*Δ* = |*τ* – 0°| (*cis*).*Δ* approaches zero as the molecular geometry approaches planarity in correspondence with a given CC bond around which the dihedral angle can be defined. In Fig. 5 we report the values of *Δ* as a function of the position of the CC bonds *R*_*i*_ along the molecular edge of HBC-Cl. Through eqn (2) each *Δ*_*i*_ is obtained from the corresponding dihedral *τ*_*i*_, which is defined in terms of the sequence of CC bonds (*R*_*i*–1_,*R*_*i*_,*R*_*i*+1_). The maximum deviations from planarity, by almost 45°, are found in correspondence with the CC bonds connecting two consecutive chlorinated aryl moieties along the molecular edge. This is expected based on the three dimensional representation of the molecule in (ud)_3_ conformation (see Fig. 3). Furthermore, the low *Δ* values of the three dihedral angles defined for each chlorinated aryl unit (close to 2°) confirm the validity of our initial assumption of considering them as locally planar in order to simplify the description of the molecular conformations of HBC-Cl.

**Fig. 6:**
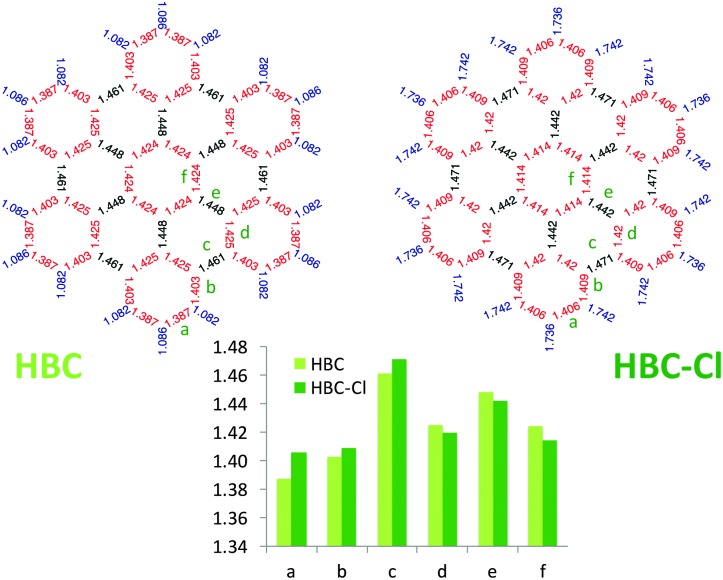
Comparison of the equilibrium bond lengths in HBC and HBC-Cl ((ud)_3_ conformer) computed using DFT.

Interestingly, as shown in Fig. 6, perchlorination slightly affects the CC bond lengths, without altering the basic pattern based on the Clar structure formed by seven aromatic sextets.^13,14^ In particular, adopting the label scheme proposed in Fig. 6, we notice that upon perchlorination the inner bonds d, e and f become shorter, while the outer bonds a, b and c become longer. Finally, it is worth mentioning that interesting non-planar structures have been also reported for fluorinated PAHs based on a coronene core.^15^ These structures are distorted to a degree similar to the case of HBC-Cl here investigated.

### 2.2 IR spectroscopy of HBC-Cl

**Fig. 7:**
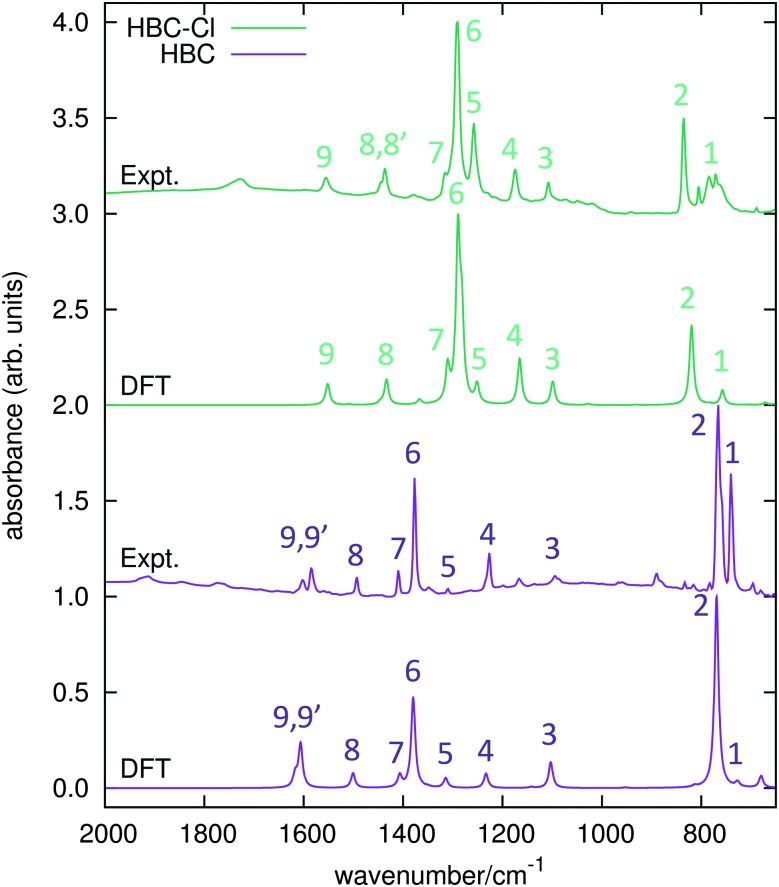
IR absorption spectra of HBC and HBC-Cl. Wavenumbers computed by DFT have been scaled by 0.98.

The IR spectra of both HBC and HBC-Cl have been measured in the solid state and compared with results from DFT calculations carried out on HBC and on the lowest energy conformation of HBC-Cl (see Table 1 and the experimental and computational methods for further details). The principal IR features found in the range between 650 and 2000 cm^–1^ have been labeled from 1 to 9 and assigned to the corresponding IR transitions predicted by DFT calculations (see Fig. 7 and Table 3). Compared with HBC-Cl, HBC presents a similar number of IR signatures. We observe a satisfactory agreement between theory and experiments, which allows us to propose the following assignment of selected IR features, based on the analysis of the nuclear displacements computed for each normal mode (see ESI,† for further details and animations of selected modes).

**Table 3:** List of observed and computed (unscaled) IR features in HBC and HBC-Cl

Feature #	Wavenumber (DFT)	Wavenumber (expt.)
HBC-Cl	*D*_3d_, (ud)_3_	
1	773 (E_u_)	771
2	836 (E_u_)	836
3	1121 (A_2u_)	1108
4	1189 (E_u_)	1175
5	1277 (E_u_)	1258
6	1308 (E_u_), 1316 (E_u_)	1291
7	1337 (A_2u_)	1315
8	1463 (E_u_), 1475 (A_2u_)	1437, 1444
9	1583 (E_u_)	1555

HBC	*D*_6h_	
1	742 (A_2u_)	740
2	784 (A_2u_)	766
3	1125 (E_1u_)	1094
4	1259 (E_1u_)	1227
5	1341 (E_1u_)	1310
6	1408 (E_1u_)	1377
7	1435 (E_1u_)	1409
8	1531 (E_1u_)	1493
9	1639 (E_1u_), 1650 (E_1u_)	1585, 1602

Band 2 in HBC is assigned to the collective out-of-plane bending of all CH bonds and correlates with the characteristic TRIO features of PAHs.^16–18^ However, the IR feature 2 of HBC-Cl is assigned to a doubly degenerate mode involving the out-of-phase C–Cl stretching of the bonds at 1 and 3 of the chlorinated aryl moieties (see Fig. 2).

Band 3 in both molecules is assigned to a collective ring-breathing mode of the aromatic core which mainly involves the six outer Clar rings; this occurs with an alternated pattern in HBC-Cl, but in HBC half of the molecule vibrates out-of-phase with respect to the other half and the mode is degenerate.

Band 6 is assigned to two closely located degenerate modes in both molecules. In both cases the pattern of the nuclear displacements is complex and mainly involves the CC bonds of the aromatic core. In HBC the normal mode is coupled with in-plane CH bending.

Band 9 is assigned in both molecules to a collective doubly degenerate ring stretching vibration, whose pattern is close to that found in discussing the Raman G line of PAHs.^19^

### 2.3 Raman spectroscopy of HBC-Cl

**Fig. 8:**
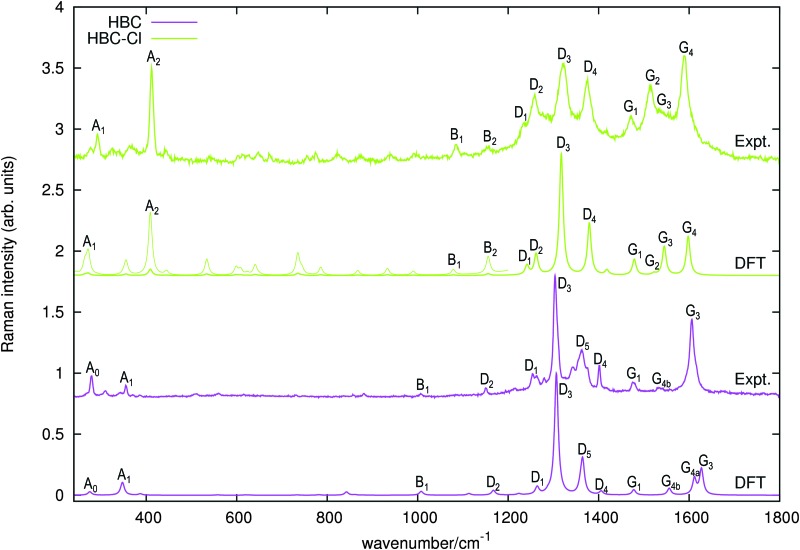
Raman spectra of HBC and HBC-Cl excited with 458 nm and 325 nm laser lines, respectively. The simulated Raman spectra computed using DFT (off-resonance) are displayed below each experimental spectrum. For HBC-Cl just the (ud)_3_ conformation has been considered in simulating the Raman spectrum. Wavenumbers computed by DFT have been scaled by 0.98. For HBC-Cl the lower wavenumber side of the simulated spectrum is also displayed with enhanced intensity (10×) to help the assignment of the experimental features. This intensity mismatch in the simulation is due to limitations in the treatment of resonance effects in the standard implementation of Raman scattering currently available in Gaussian09.^20^

The Raman spectra of HBC and HBC-Cl are reported in Fig. 8. The two molecules display a similar spectral pattern, dominated by features which have been attributed to G and D modes in HBC.^19,21^ The analysis of the nuclear displacements computed by DFT for the G and D Raman lines displays typical and recognizable patterns which can be put in correspondence between HBC and HBC-Cl.

Following the labeling scheme adopted in Fig. 8, below we discuss and compare the nuclear displacement patterns of selected modes of HBC and HBC-Cl which are associated with relatively intense experimental Raman lines. The complete list of modes is reported in Table 4 and further details are given in the ESI.†

**Table 4:** List of observed and computed (unscaled) Raman features in HBC-Cl and HBC. The G and D features of HBC have also been discussed previously.^21^ Experimentally feature G_2_ is unresolved because it is very close to the strong G_3_ feature, while feature G_4a_ is too weak to be observed

Feature #	Wavenumber (DFT)	Wavenumber (expt.)
HBC-Cl	*D*_3d_, (ud)_3_	
A_1_	276 (A_1g_)	292
A_2_	415 (A_1g_, E_g_)	412
B_1_	1095 (A_1g_)	1086
B_2_	1173 (E_g_)	1157
D_1_	1259 (E_g_)	1235
D_2_	1280 (A_1g_)	1259
D_3_	1336 (A_1g_)	1321
D_4_	1399 (A_1g_)	1376
G_1_	1500 (E_g_)	1470
G_2_	1544 (A_1g_)	1514
G_3_	1567 (E_g_)	1536
G_4_	1621 (E_g_)	1589

HBC	*D*_6h_	
A_0_	279 (E_2g_)	279
A_1_	352 (A_1g_)	355
B_1_	1022 (A_1g_)	1007
D_1_	1282 (E_2g_)	1255
D_2_	1184 (A_1g_)	1150
D_3_	1325 (A_1g_)	1304
D_4_	1426 (A_1g_)	1402
D_5_	1384 (A_1g_)	1363
G_1_	1499 (E_2g_)	1476
G_2_	1647 (A_1g_)	—
G_3_	1650 (E_2g_)	1606
G_4a_	1635 (E_2g_)	—
G_4b_	1578 (E_2g_)	1533

**Fig. 9:**
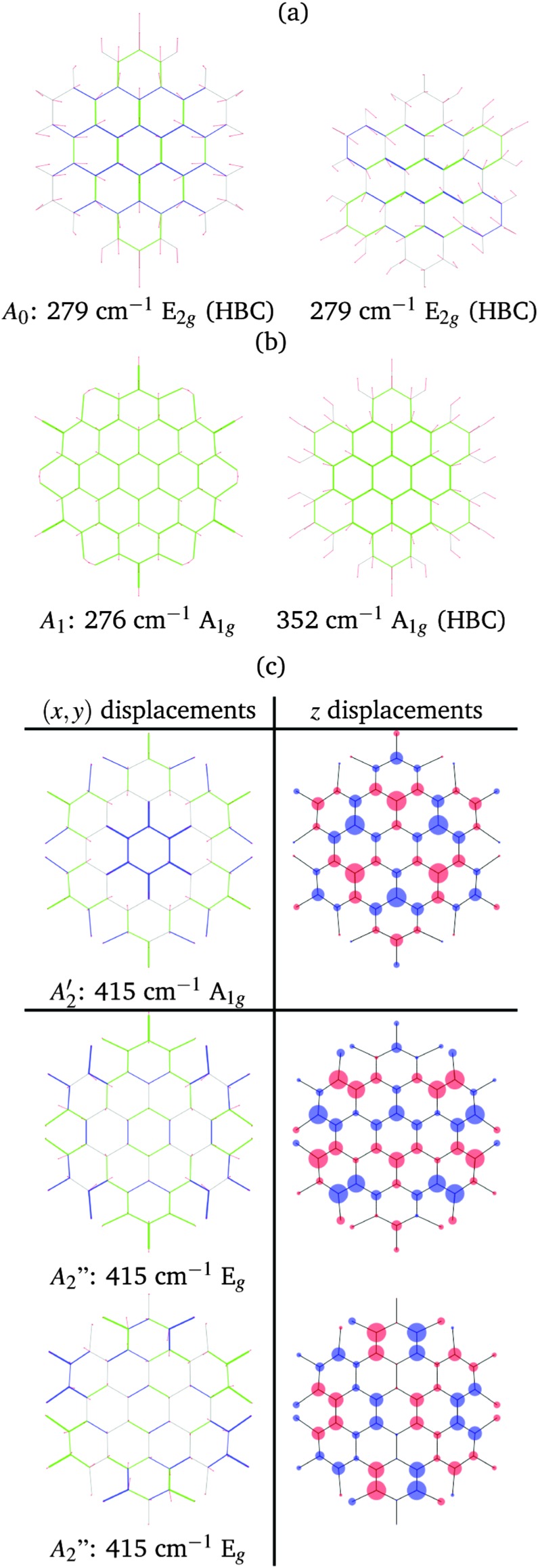
Representation of the low wavenumber (A region) normal modes of HBC and HBC-Cl relevant for Raman spectroscopy according to DFT calculations: (a) the A_0_ doubly degenerate mode of HBC; (b) the compared breathing modes of HBC-Cl and HBC; (c) the degenerate A_1g_ and E_g_ modes computed at 415 cm^–1^. For in-plane modes red lines represent displacement vectors; CC bonds are represented as green (blue) lines of different thicknesses according to their relative stretching (shrinking). For out-of-plane modes the size of blue/red circles of the molecular sketch is proportional to nuclear displacements in the *z* direction.

In both HBC and HBC-Cl the mode A_1_ is assigned to the in-phase collective breathing of the molecule along a radial direction (Fig. 9b). The collective breathing of HBC (feature A_1_, observed at 355 cm^–1^) is significantly red-shifted in HBC-Cl (feature A_1_, observed at 292 cm^–1^). This is due to the mass effect of the heavy chlorine atoms at the molecular edge. Interestingly, the A_0_ feature observed in HBC (assigned to a doubly degenerate mode which involves mainly the CC stretching of the aromatic core – see Fig. 9a) is characteristic of HBC: no similar mode is computed or observed in HBC-Cl.

In the low wavenumber region we find in the experimental spectrum of HBC-Cl a strong Raman line at 412 cm^–1^ (A_2_) which does not find a counterpart in HBC. Based on DFT calculations, this corresponds to three very close Raman active modes computed at 415 cm^–1^ (see Fig. 9c) which arise from the degeneracy of an A_1g_ mode with a doublet of E_g_ species. These modes are characterized by vibrational displacements which have a sizable contribution both along the *z*-axis and within the (*x*,*y*) plane. DFT calculations predict a significantly stronger intensity for the A_1g_ mode than the degenerate E_g_ doublet. Hence it is reasonable to associate the experimental A_2_ feature mainly with the totally symmetric mode represented in Fig. 9c; looking at the (*x*,*y*) representation of this A_1g_ mode, one recognizes the breathing pattern of the 7 inner rings (which could be associated with a coronene moiety) in the center of HBC-Cl. During this vibration the outer part of the molecule breathes out-of-phase with respect to the center. As for the E_g_ degenerate doublet, the associated nuclear displacements shown in Fig. 9c display a collective pattern characterized by alternated out-of-plane displacements along the *z*-direction accompanied by displacements in the (*x*,*y*) plane which are mostly localized at the molecular periphery.

**Fig. 10:**
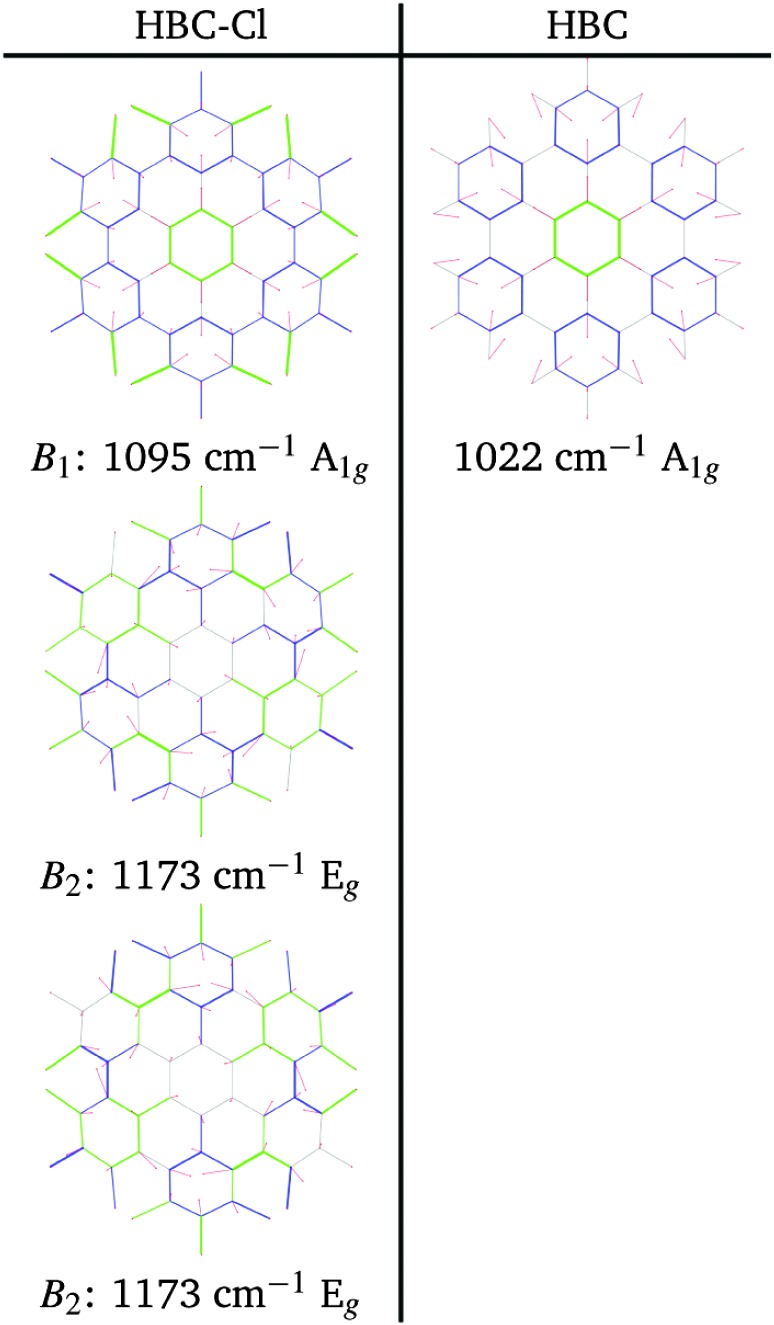
Representation of the normal modes of HBC-Cl relevant for Raman spectroscopy in the B region according to DFT calculations.

At a higher wavenumber, in the region located between the A-modes and the D-band modes we find Raman active modes which are described as collective breathing vibrations of the Clar rings which can be identified in the molecular structure (see Fig. 10 – further details in the ESI†). Similar modes have been also identified in a previous study on the graphene molecule C78.^22^

**Fig. 11:**
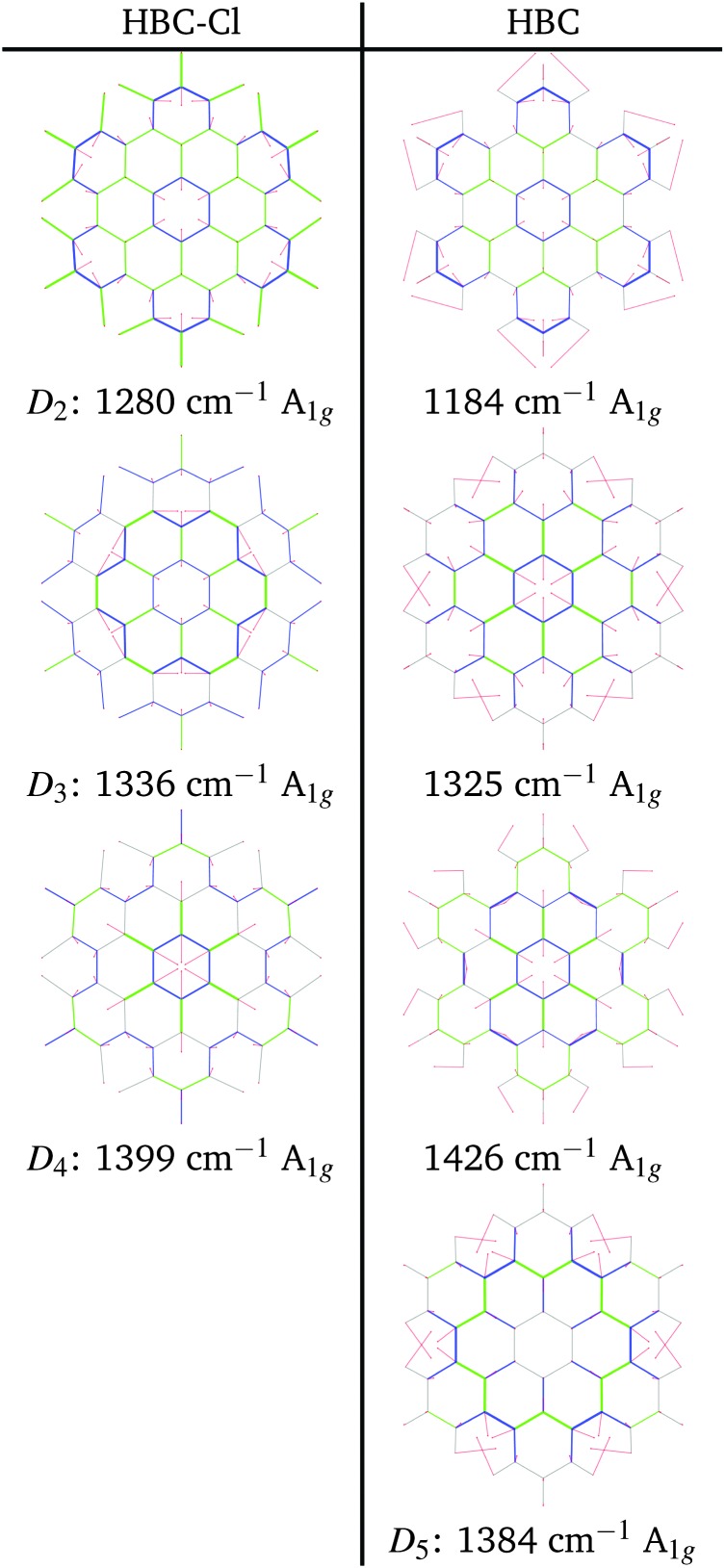
Representation of the normal modes of HBC and HBC-Cl relevant for Raman spectroscopy in the D region according to DFT calculations.

At progressively higher wavenumbers one finds modes which can be related to the pattern expected for D-modes (Fig. 11).^19^ The Raman feature D_2_ is assigned to the ring breathing of the central Clar ring, out of phase with respect to the stretching of the radial CC bonds which emanate from the central Clar ring. It partially displays the canonical D-mode pattern,^19^ the notable exception being the wrong phase of the stretching of the bonds of kind d (see Fig. 6). In HBC-Cl the D_2_ mode is coupled with the in-phase C–Cl stretching of the chlorinated aryl moieties. In HBC the D_2_ mode is coupled with the collective in-plane bending of the CH bonds at positions (1,3) (see Fig. 2). D_2_ is notably blue-shifted in HBC-Cl compared to HBC (expt. 109 cm^–1^).

The strong Raman active line D_3_ is assigned to the in-phase ring breathing of all the seven Clar rings coupled with the CC shrinking of the bonds of kinds c and e (see Fig. 6). This is the mode which fully displays the expected D-mode pattern.^19^ In HBC the D_3_ mode is coupled with the collective in-plane bending of the CH bonds at positions 1 and 3 (see Fig. 2). Interestingly, comparing modes D_2_ and D_3_ in HBC, one finds that the relative phase between the inner Clar ring breathing and the outer CH-bending inverts. Hence the modes D_2_ and D_3_ of HBC can be approximately described as the doublet arising from the vibrational coupling between the collective breathing coordinate of the seven Clar rings and the collective CH bending of the CH bonds at positions 1 and 3. Mode D_4_ is assigned to the breathing of the central Clar ring, out-of-phase with respect to the approximate breathing of the outer six Clar rings. In HBC the D_4_ mode is coupled with the collective in-plane bending of the CH bonds at 1 and 3 (see Fig. 2) while in HBC-Cl the D_4_ mode is coupled with the collective stretching of the C–Cl bonds at 2 (see Fig. 2). Finally, feature D_5_ is found just in HBC and is assigned to the collective CC stretching at the edge of the molecule, mainly at bonds of kinds c and d (Fig. 6) coupled with the collective in-plane CH bending at positions 1 and 3 (see Fig. 2).

The G-modes appear in the next wavenumber region, above the D-modes. In graphene molecules the G-modes display collective displacement patterns^19^ which can be associated with those of the ν_16_ E_2g_ ring-stretching mode of benzene.^23^

**Fig. 12:**
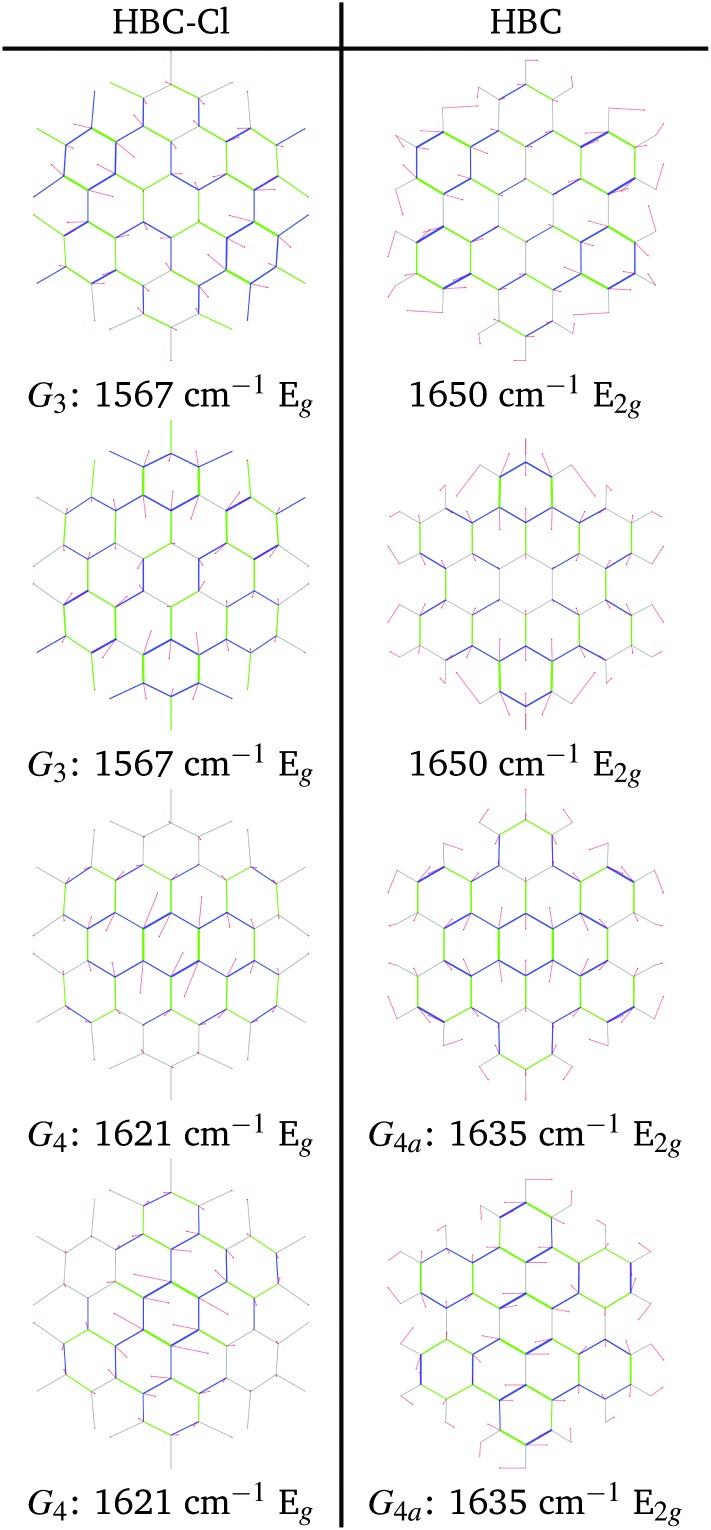
Representation of the normal modes of HBC and HBC-Cl relevant for Raman spectroscopy in the G region according to DFT calculations.

The G_3_ Raman feature of HBC is assigned to a doubly degenerate mode which involves CC stretching mainly in the outer part of the molecule and it is coupled with collective in-plane CH bending at positions 1 and 3 (see Fig. 2). As observed in Fig. 12, the G_3_ feature of HBC-Cl is assigned to a doubly degenerate mode with a similar pattern to HBC. However, compared to HBC, the G_3_ mode of HBC-Cl is remarkably red-shifted (expt. 70 cm^–1^). Finally, the G_4_ feature of HBC-Cl is assigned to a doubly degenerate mode which involves the ring stretching of the three central rings next to each other along a row. In HBC there are two doubly degenerate modes with a similar nuclear displacement pattern which have been named G_4a_ and G_4b_. They both involve ring stretching vibrations coupled with a collective in-plane CH bending.

## 3 Conclusions

Out-of-plane distortions in molecular models of graphene are not seriously impairing the π-conjugation: this is supported by DFT calculations on perchlorinated HBC which shows a markedly non-planar equilibrium structure due to the steric hindrance of Cl atoms at the molecular edge, which is in agreement with the structure obtained from the single-crystal X-ray analysis. Both the computed HOMO–LUMO gap and the vibrational properties observed using Raman and IR spectroscopies show that HBC-Cl possesses a π-conjugation similar to HBC. Interestingly, even in the non-planar case of HBC-Cl, the π-stacking, which is a crucial property for charge transport in molecular electronic devices based on HBC,^7,24^ is still possible due to specific steric interactions and interlocking of chlorine hindrances, as depicted in Fig. 1. Vibrational spectroscopy complemented with DFT calculations proves to be informative about the chemical structure of HBC and HBC-Cl: distinct markers can be directly associated with specific moieties. For instance, the TRIO marker in the IR of HBC (766 cm^–1^) is due to the symmetric hydrogen-terminated molecular edge, while the persistence of the strong D_3_ peak in HBC (1304 cm^–1^) and HBC-Cl (1321 cm^–1^) proves the similar π-conjugated nature of the two molecules, independent of planarity. A thorough analysis of the different stable conformers of HBC-Cl, carried out by geometry optimization using DFT methods, revealed that a rich variety of structures, including chiral enantiomers, can be obtained by suitable chemical substitution at the edges of HBC. While, in the case under study, the lowest energy conformation with achiral (ud)_3_ conformation is the structure found in the crystal,^3^ we can infer that the introduction of selected edge substituents and/or synthetic pathways could give rise to novel structures, for instance based on propeller-shaped chiral units. These could exhibit appealing chiroptical properties, similar to the class of helicenes.^25^

## 4 Experimental and computational methods

Samples of HBC and HBC-Cl were synthesized as described in ref. 3 and 26, respectively. Micro-FT-IR measurements on all the molecules were carried out using Nicolet Nexus equipment coupled with a Thermo-Nicolet Continuμm infrared microscope and a cooled MCT detector (77 K). The spectra of the samples (as powders) were acquired by using the diamond anvil cell technique with a 15× infrared objective (64 scans, 1 cm^–1^ resolution). Compared with the KBr pellet technique, the micro-FT-IR setup allows recording spectra with a minimal sample amount. The micro-Raman measurements reported in this work have been carried out using Jobin-Yvon Labram HR800UV equipment using laser excitations at 458 nm and 325 nm, which have been selected to optimize the Raman signal and keep the fluorescence background as low as possible. All DFT calculations reported in this work have been carried out using Gaussian09^20^ by adopting the B3LYP functional and the 6-31G(d,p) basis set. The computer rendered representations of the molecular models reported in Fig. 3 and 1 have been obtained using the program YASARA.^27^ The representation of the molecular orbitals reported in Fig. 4 has been obtained using the open source program Avogadro (version 1.1.1).^28^ A set of post-processing programs developed at Politecnico di Milano has been used to generate the representation of the vibrational normal modes and simulate the Raman and IR spectra from the results of DFT calculations.
