# Supplementary material for: Edge chlorination of hexa-peri-hexabenzocoronene investigated by density functional theory and vibrational spectroscopy
Source: Phys Chem Chem Phys. 2016 Feb 25;18(17):11869–78. doi: 10.1039/c5cp07755a (PMC4906847; doi:10.1039/c5cp07755a)
Supplement: Supplementary file 1 [file CP-018-C5CP07755A-s001.pdf]

Electronic Supplementary Material (ESI) for Physical Chemistry Chemical Physics.
This journal is © the Owner Societies 2016

Edge chlorination of hexa-peri-hexabenzocoronene investigated by density functional theory and vibrational spectroscopy

Ali Maghsoumi[a], Akimitsu Narita[b], Renhao Dong[c], Xinliang Feng[c], Chiara Castiglioni[a], Klaus Müllen[b], and Matteo Tommasini[a]

[a] Dipartimento di Chimica, Materiali e Ingegneria Chimica – Politecnico di Milano, Piazza Leonardo da Vinci, 32 – 20133 Milano, Italy
[b] Max Planck Institute for Polymer Research, Ackermannweg 10, D-55128, Mainz, Germany
[c] Center for Advancing Electronics Dresden (CFAED) & Department of Chemistry and Food Chemistry, Dresden University of Technology, Walther-Hempel-Bau Mommsenstrasse 4, 01062 Dresden, Germany

Electronic Supplementary information (ESI)

Contents

S1. Description of normal modes of HBC and HBC-Cl relevant for IR and Raman

S2. Animations of selected vibrational normal modes of HBC and HBC-Cl relevant for IR

S1.1 Description of normal modes of HBC and HBC-Cl relevant for IR

We follow here the notation of the experimental features given in Figure 7 of the main text.

HBC-Cl

- 1 is assigned to a degenerate pair of collective out-of-plane bending modes of the aromatic core; considering a given ring of the core, these modes involve the six carbon atoms with an alternated pattern of up/down displacements.

- 2 is assigned to a doubly degenerate mode involving the out-of-phase C-Cl stretching of the bonds at 1 and 3 (see Figure 2 of the main text) of the chlorinated aryl moieties.

- 3 is assigned to a collective ring-breathing mode of the aromatic core; this occurs with an alternating pattern and mainly involves the six outer Clar rings of HBC-Cl.

- 4 is assigned to a doubly degenerate mode which mainly involves the in-phase C-Cl stretching of the bonds at 1 and 3 (see Figure 2 of the main text) of the chlorinated aryl moieties.

- 5 is assigned to a doubly degenerate mode which mainly involves the CC stretching of the bonds which could be thought to form the edge of a coronene moiety inscribed in HBC-Cl aromatic core (*i.e.*, bonds of kind c and d in Figure 6 of the main text).

- 6 is assigned to two closely located degenerate modes, computed at 1308 cm^{-1} and 1316 cm^{-1}. In both cases the pattern of nuclear displacements is complex and mainly involves the CC bonds of the aromatic core.

- 7 is assigned to the in-phase stretching of the three C-Cl bonds of each chlorinated aryl moiety; the pattern of the mode alternates along HBC-Cl edge (*i.e.*, three aryl moieties have shrinking C-Cl bonds while the other three have stretching C-Cl bonds).

- 8 is assigned to two closely located modes (1463 cm^{-1}, E_u and 1475 cm^{-1}, A_{2u}). The two peaks are not well resolved and appear as a structured feature both in the experimental and simulated spectrum. The E_u normal modes collectively involve CC bonds of the aromatic core; the A_{2u} mode displays a more recognizable pattern, with bonds of kind e (see Figure 6 of the main text) which alternatively stretch in an out-of-phase fashion.

- 9 is assigned to a collective doubly degenerate ring stretching vibration (computed at 1583 cm^{-1}), whose pattern is close to that found in discussing the G line in Raman spectroscopy of PAHs.[1]

HBC

- 1 is assigned to an alternated out-of-plane bending vibration of the carbon atoms in the aromatic core, coupled with the in-phase out-of-plane bending of the CH bonds at 1,3 (see Figure 2 of the main text).

- 2 is assigned to the collective out-of-plane bending of all CH bonds and correlates with the characteristic TRIO features of PAHs.[2,3,4]

- 3 is a degenerate doublet involving a collective ring-breathing of the six outer Clar rings of HBC, with half the molecule vibrating out-of-phase with respect to the other half.

- 4,5,6,7,8 are assigned to degenerate modes involving CC stretching of the aromatic core, coupled with in-plane CH bending.

- 9 is assigned to collective doubly degenerate ring stretching vibrations, whose pattern is close to that found in discussing the G line in Raman spectroscopy of PAHs.[1]

S1.2 Description of the normal modes of HBC and HBC-Cl relevant for Raman

We follow here the notation of the experimental features given in Figure 8 of the main text.

S1.2 Raman B modes

- Peak B_1 is assigned to the ring-breathing mode of the central Clar ring of HBC and HBC-Cl which vibrates out-of-phase with respect to the six outer Clar rings. In HBC-Cl mode B_1 is coupled with the collective C-Cl stretching of the chlorinated aryl moieties. In particular C-Cl stretching at (1, 3) (see Figure 2 of the main text) are both out-of-phase with respect to C-Cl stretching at 2 (see Figure 2 of the main text). Compared to HBC, this mode is remarkably blue-shifted in HBC-Cl (experimentally by 79 cm^{-1}, while according to DFT by 73 cm^{-1}).

- Feature B_2 is only found in the HBC-Cl molecule and it is assigned to a doubly degenerate mode which involves the CC stretching of the outer part of the molecule (mainly bonds of kind *c* and *d*, see Figure 6 of the main text) coupled with the C-Cl stretching of the chlorinated aryl moieties.

S1.2 Raman D and G modes

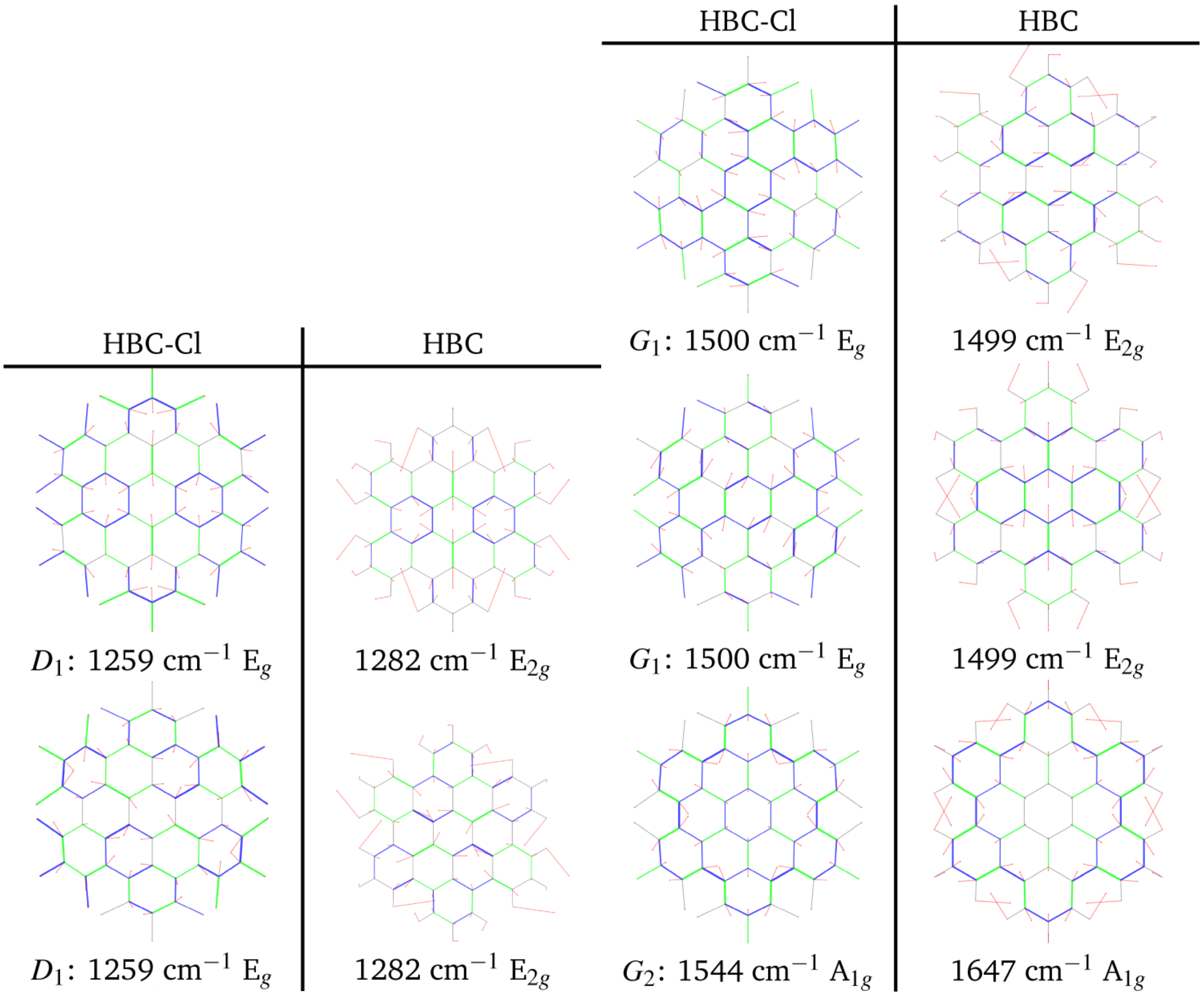

Figure S1.2 nuclear displacements of the D and G modes of HBC and HBC-Cl discussed here.

- The weak Raman active doubly degenerate mode D_1 is assigned to a vibration which involves the breathing of four Clar rings in both HBC and HBC-Cl. In HBC-Cl the D_1 mode is coupled with the in-phase C-Cl stretching of the chlorinated aryl moieties. In HBC the D_1 mode is coupled with the collective in-plane bending of the CH bonds at positions (1,3) (see Figure 2 of the main text).

- The experimental G_1 feature of HBC and HBC-Cl (see Figure 8 of the main text) is assigned to doubly degenerate modes of E species which mainly involve the CC stretching of bonds in the center of the molecule. In HBC the mode G_1 is coupled with collective in-plane CH bending.

- Feature G_2 is assigned to a totally symmetric mode which involves CC stretching (*i.e.*, ring stretching – see above) mainly at the outer rings of the molecule (bonds of kind *b* in Figure 6 pf the main text). In HBC the mode G_2 is coupled with collective in-plane CH bending at positions (1,3) (see Figure 2 of the main text). In HBC-Cl the G_2 mode involves the collective C-Cl stretching of bonds at 2 (see Figure 2 of the main text) and it is significantly red-shifted compared to HBC (according to DFT by 103 cm^{-1}; the experimental determination of the position of G_2 for HBC is not feasible because it is a weak mode overlapped with other stronger G components).

S2. Animations of selected vibrational normal modes of HBC and HBC-Cl relevant for IR

An archive file (7-Zip format [5]) is included in the ESI. It contains a set of animated gif files associated to the modes of HBC and HBC-Cl listed in Tables 3 and 4. These animations have been obtained with the program ChemCraft [6].

References

[1] C. Castiglioni, M. Tommasini and G. Zerbi, Philosophical Transactions of the Royal Society of London Series A-Mathematical Physical and Engineering Sciences, 2004, **362**, 2425–2459.

[2] M. Tommasini, A. Lucotti, M. Alfè, A. Ciajolo and G. Zerbi, Spectrochimica Acta Part A: Molecular and Biomolecular Spectroscopy, 2016, **152**, 134 – 148.

[3] A. Centrone, L. Brambilla, T. Renouard, L. Gherghel, C. Mathis, K. Mullen and G. Zerbi, Carbon, 2005, **43**, 1593–1609.

[4] M. Von Zander and B. G. Teubner, Angewandte Chemie, 1996, **108**, 2411–2411.

[5] 7-Zip: see website http://www.7-zip.org (accessed 10 Feb 2016).

[6] ChemCraft: see website http://www.chemcraftprog.com/index.html (accessed 10 Feb 2016).
